# Short Residence Times of DNA-Bound Transcription Factors Can Reduce Gene Expression Noise and Increase the Transmission of Information in a Gene Regulation System

**DOI:** 10.3389/fmolb.2020.00067

**Published:** 2020-04-28

**Authors:** Eugenio Azpeitia, Andreas Wagner

**Affiliations:** ^1^Department of Evolutionary Biology and Environmental Studies, University of Zürich, Zurich, Switzerland; ^2^Swiss Institute of Bioinformatics, Lausanne, Switzerland; ^3^Centro de Ciencias Matemáticas, UNAM, Morelia, Mexico; ^4^Santa Fe Institute, Santa Fe, NM, United States

**Keywords:** residence time, transcription factor-DNA interaction, regulation of gene expression, gene expression noise, Information acquisition, stochastic processes, computational modeling and simulations

## Abstract

Gene expression noise is not just ubiquitous but also variable, and we still do not understand some of the most elementary factors that affect it. Among them is the residence time of a transcription factor (TF) on DNA, the mean time that a DNA-bound TF remains bound. Here, we use a stochastic model of transcriptional regulation to study how residence time affects the gene expression noise that arises when a TF induces gene expression. We find that the effect of residence time on gene expression noise depends on the TF’s concentration and its affinity to DNA, which determine the level of induction of the gene. At high levels of induction, residence time has no effect on gene expression noise. However, as the level of induction decreases, short residence times reduce gene expression noise. The reason is that fast on-off TF binding dynamics prevent long periods where proteins are predominantly synthesized or degraded, which can cause excessive fluctuations in gene expression. As a consequence, short residence times can help a gene regulation system acquire information about the cellular environment it operates in. Our predictions are consistent with the observation that experimentally measured residence times are usually modest and lie between seconds to minutes.

## Introduction

All gene expression is noisy. It produces mRNA and protein molecules whose numbers fluctuate randomly. Such noise is caused by stochastic molecular interactions, which include interactions between transcription factors (TFs) and DNA, and by the stochastic synthesis and degradation of molecules (Kepler and Elston, 2001; Raj and van Oudenaarden, 2009). Gene expression noise affects multiple biological processes. For example, it can promote phenotypic diversity, influence the coordination of gene expression, trigger cell differentiation, and facilitate evolutionary transitions (Kepler and Elston, 2001; Fraser and Kaern, 2009; Raj and van Oudenaarden, 2009; Eldar and Elowitz, 2010; Sanchez et al., 2013; Karig et al., 2018; Urban and Johnston, 2018). Furthermore, noise can also reduce a cell’s ability to acquire information about its environment. Such information is essential whenever cells need to respond to changing environments (Bowsher and Swain, 2014; Levchenko and Nemenman, 2014). Information is acquired by signaling pathways that modulate the activity or concentration of TFs, which up-regulate or down-regulate effector genes. Thus, reducing gene expression noise can increase the ability of a regulated gene to capture information about a TF’s changing concentration or activity, which is fundamental to produce an optimal cellular response to environmental change (Rhee et al., 2012).

Gene expression regulation is being studied by many researchers whose insights improve our capacity to control and modify living systems (Li et al., 2007; Lelli et al., 2012; Sainsbury et al., 2015; Pope and Medzhitov, 2018). However, we still do not fully understand how some elementary properties of the interaction of a TF with its binding site on DNA affect the stochastic dynamics of gene expression and the acquisition of information (Einav and Phillips, 2019). One of these properties is a TF’s residence time on its DNA binding site – the mean time that a TF remains bound to DNA. The residence time is equal to the inverse of the dissociation rate *k*_*d*_ between a TF and DNA. Theoretical and experimental work has shown that the dissociation rate can affect gene expression, affect the size of gene expression bursts (Skupsky et al., 2010; Kumar et al., 2015; Fujita et al., 2016; Donovan et al., 2019), and modulate gene expression noise (Peccoud and Ycart, 1995; Raser, 2004; Iyer-Biswas et al., 2009; Grönlund et al., 2013). However, it is difficult to discern whether the dissociation rate affects gene expression by altering the residence time or the affinity between a TF and DNA, because both depend on the dissociation rate *k*_*d*_ (affinity is given by the ratio *K_*eq*_* = *k_*d*_/k_*a*_* [M], where *k*_*a*_ is association rate between a TF and DNA).

Many TFs bind DNA transiently, with residence times ranging between seconds and minutes (Hager, 2009; Geertz et al., 2012; Mazza et al., 2012; Gebhardt et al., 2013; Mueller et al., 2013; Chen et al., 2014; Morisaki et al., 2014; Sugo et al., 2015; Deluz et al., 2016; Clauß et al., 2017; Liu and Tjian, 2018; Rullan et al., 2018). Such TFs include MYC, p53, and glucocorticoid receptors, which are involved in fundamental processes such as apoptosis, DNA repair, DNA maintenance, and stress responses. They also include pioneer TFs that directly interact with chromatin and open it (Oakley and Cidlowski, 2013; Joerger and Fersht, 2016; Swinstead et al., 2016). The duration of a TF’s residence time on a specific binding site can vary, even for different tissues within the same organisms, by chromatin modifications and the interaction of the TF with other molecular components (Giglia-Mari et al., 2009; van Werven et al., 2009; Mueller et al., 2013). Such variation implies that residence time may play a role in regulating gene expression. Nevertheless, we do not know how residence time affects gene expression noise, because of limitations in experimental technology. For example, it is difficult to measure residence time and simultaneously quantify the rate of gene expression. It is also hard to quantify the number of TF binding events at a specific binding site in a given time (Mueller et al., 2013; Liu and Tjian, 2018; Donovan et al., 2019). Moreover, it is challenging to experimentally modify only residence time without also affecting affinity.

Here we circumvent these limitations through a stochastic model of a TF that induces gene expression by binding to DNA. With this model, we study how residence time affects gene expression noise and the amount of information acquired by a gene expression system. Our analyses show that the effect of residence time increases as the level of induction of a gene decreases. At high induction levels, residence time has no effect on gene expression. However, as induction levels decrease, shorter residence times reduce the amount of gene expression noise and produce more regular gene expression dynamics. Shorter residence times also increase a gene regulation system’s capacity to acquire information about the concentration of a TF. In sum, shorter residence times improve a gene’s response to changes in its cellular environment.

## Results

### Model and Main Concepts

**FIGURE 1:**
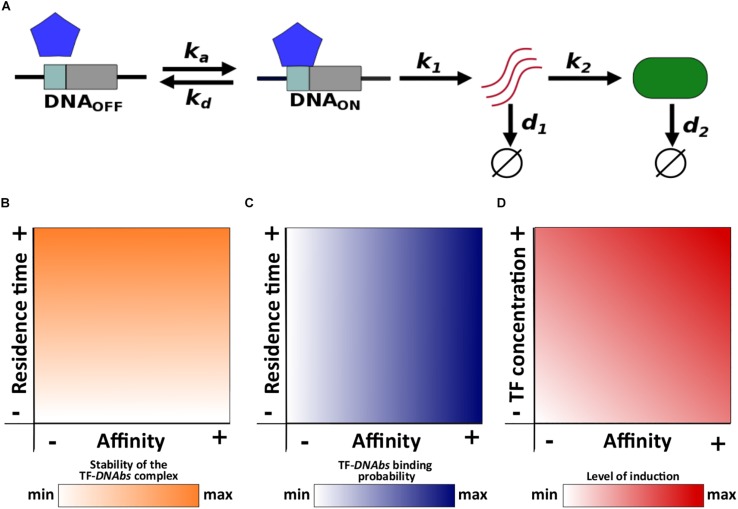
Schematic description of the model and main concepts. **(A)** *k*_*a*_ and *k*_*d*_ correspond to the association and dissociation rate, respectively; *k*_1_ and *k*_2_ correspond to the mRNA and protein synthesis rate, respectively; *d*_1_ and *d*_2_ correspond to the mRNA and protein degradation rate, respectively. Relationships of both residence time and affinity with **(B)** the stability of the TF-*DNA*_*bs*_ complex, and **(C)** TF-*DNA*_*bs*_ binding probability. **(D)** Relationship of affinity and TF concentration with the level of induction.

We use a two-state model of gene expression that represents the transcriptional activation and inactivation of a gene. In this model, TF molecules associate and dissociate from the gene’s TF binding site (*DNA*_*bs*_) at rates *k*_*a*_ (M^–1^s^–1^) and *k*_*d*_ (s^–1^), respectively. The regulated gene is expressed only when the binding site is bound by a TF (i.e., the TF activates gene expression), in which case the gene is transcribed into mRNA at rate *k*_1_. The resulting mRNA is then translated into protein molecules at rate *k*_2_. Finally, mRNA and protein molecules degrade at rates *d*_1_ and *d*_2_, respectively (Figure 1A).

Residence time is the average life span or half-life (*t*_1/2_) of the TF-DNA_*bs*_ complex. In other words, residence time quantifies the stability of this complex (Figure 1B). Affinity is quantified with the equilibrium constant *K*_*eq*_. The equilibrium constant is equal to the concentration of free TF at which half of all binding sites are occupied. A high equilibrium constant is equal to a low affinity, because it means that a large concentration of TF is required to occupy 50% of binding sites. For a given TF concentration, the probability that a binding site is occupied increases with increasing affinity (Figure 1C).

Although both affinity and residence time depend on the dissociation rate *k*_*d*_, they can be modified independently from each other. Changing the dissociation rate will modify the residence time, but it can leave the affinity unchanged if the association rate changes appropriately to keep the ratio *k*_*d*_/*k*_*a*_ constant. Conversely, by changing only the association rate, affinity can be modified without altering residence time.

Notice that affinity and TF concentration jointly determine the level of induction of gene expression, because a gene is more likely to be active when the concentration of free TF is higher than its affinity to DNA. Conversely, when this concentration is lower than the affinity, the gene will tend to be inactive. One can increase the level of a gene’s induction by increasing either TF concentration or affinity (Figure 1D). Below we change the level of induction by modifying TF concentration, but changing affinity itself yields the same observations (see Supplementary Data 1).

We simulate gene regulation dynamics using Gillespie’s algorithm (Gillespie, 1977), which reproduces the stochastic dynamics of many chemical systems, using biologically meaningful values of all biochemical parameters (Supplementary Table 1 and Methods). Because both the mRNA and protein output of our modeled gene regulation system behave qualitatively identically, we focus on the protein output below (see Supplementary Figures for mRNA).

### Short Residence Times Reduce Gene Expression Noise and Modulate Gene Expression Dynamic

We first study the effect of residence time and affinity on gene expression noise. To quantify noise, we quantified the size of the temporal fluctuations in the number of proteins, as the difference between the maximal and the minimal number of expressed protein molecules (*NP__max__*−*N_P_min__*), and averaged this difference over 1000 simulations. Two alternative noise measures, the coefficient of variation and the Fano factor yield identical observations (see Supplementary Data 2 and Supplementary Figure 1).

In these simulations, we varied residence time within the interval [1 s, 1 h], TF concentration within the interval [10^–11^M, 10^–7^M], and set the affinity to 10^–9^M. Notice that the TF concentration interval ranges two orders of magnitude below and above the affinity, which implies that the level of gene induction ranges from almost always inactive to almost always active. Hence, high and low TF concentration values correspond to high and low induction levels, respectively.

**FIGURE 2:**
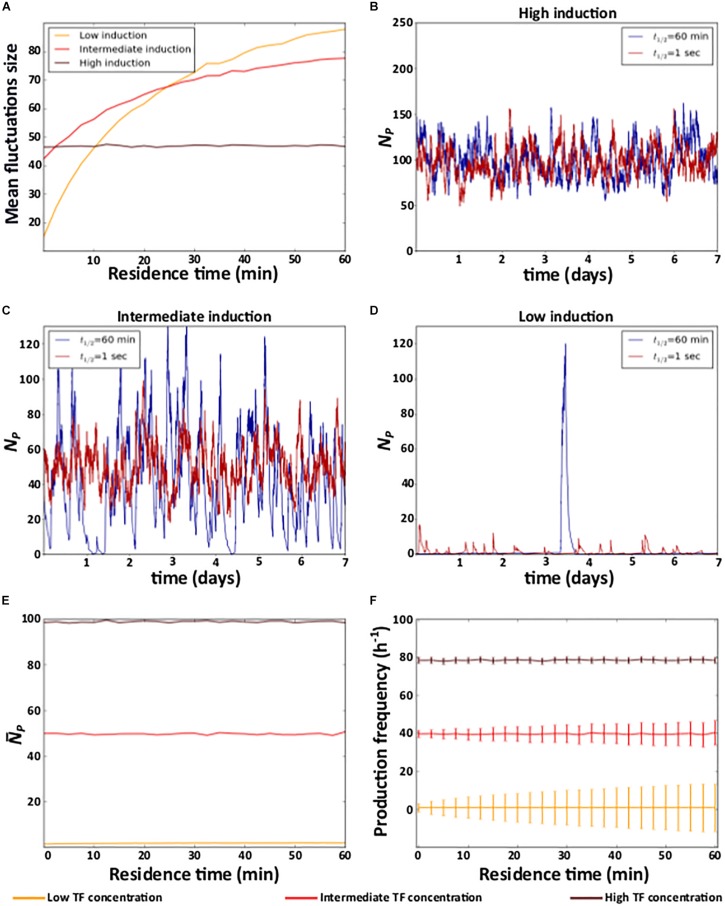
Residence time and noise. **(A)** Mean fluctuation size in the number of protein molecules (*y* axis) at different levels of induction as a function of residence time (*x* axis). **(B–D)** Example time trajectories of the number of protein molecules *N*_*P*_ at three different levels of induction. Analyses of **(E)** mean number of protein (${\bar{N}}_{P}$) **(F)** mean and coefficient of variation of the frequency of protein production events. **(B–D)** Red and blue lines show data for short (1 s) and a long (1 h) residence times, respectively. **(A,E,F)** High (TF = 10^–7^M), intermediate (TF = 10^–9^M), and low (TF = 10^–11^M) levels of induction are indicated in the color legend below the figure.

At the highest TF concentration, residence time does not affect noise (Figure 2A and Supplementary Figure 2A). However, as the TF concentration decreases, a longer residence time increases noise (Figure 2A and Supplementary Figure 2A). For example, individual protein expression trajectories at extremely short (*t*_1/2_ = 1 s) and long (*t*_1/2_ = 1 h) residence times are very similar at the highest TF concentration (Figure 2B and Supplementary Figure 2B). However, as TF concentration decreases, long residence times cause sporadic, but large fluctuations in protein expression (Figures 2C,D and Supplementary Figures 2C,D).

To understand these observations, notice that when the TF concentration is very high, the level of induction is high because a TF molecule is bound to DNA most of the time. In this case, gene expression resembles that of a constitutive gene, regardless of the TF’s residence time. In consequence, gene expression noise is only determined by the degradation and synthesis rates of mRNA and protein (Kepler and Elston, 2001; Raj and van Oudenaarden, 2009). In other words, it is independent of residence time (Figures 2A,B and Supplementary Figures 2A,B).

This is no longer true as the TF concentration decreases. In this case, the probability that a TF is bound to DNA at any one time decreases, and longer residence times increase the average amount of time that a TF is either bound or unbound. In other words, longer residence times produce longer periods of active and inactive gene expression. During active periods, proteins are produced, whereas during inactive periods, previously expressed proteins decay. Thus, longer residence times lengthen both active and inactive periods, which results in large fluctuations in the number of proteins (Figures 2C,D and Supplementary Figures 2C,D). Reducing the residence time (at constant induction) decreases the duration of both active and inactive periods by the same amounts. As a result, expressed molecules accumulate and decay for shorter time periods, and fluctuations in these molecules become smaller (Figures 2A–D and Supplementary Figures 2A–D).

In contrast to its effects on noise, residence time does not affect the mean level of protein expression, which only depends on the level of induction (Figure 2E and Supplementary Figure 2E). The reason is that the frequency of both protein production and degradation events (i.e., the mean number of proteins produced and degraded in a given period of time) is not affected by residence time, regardless of the level of induction (Figure 2F and Supplementary Figures 2F, 3A,B). However, as the level of induction decreases, shorter residence times decrease variation in the frequency of production and degradation events (Figure 2F and Supplementary Figures 2F, 3A,B). As a consequence, protein expression is more homogeneous at shorter residence times, because protein production and degradation events alternate more regularly, except at the very lowest affinities (Supplementary Data 3 and Supplementary Figures 3C–F).

### Residence Time and Information

**FIGURE 3:**
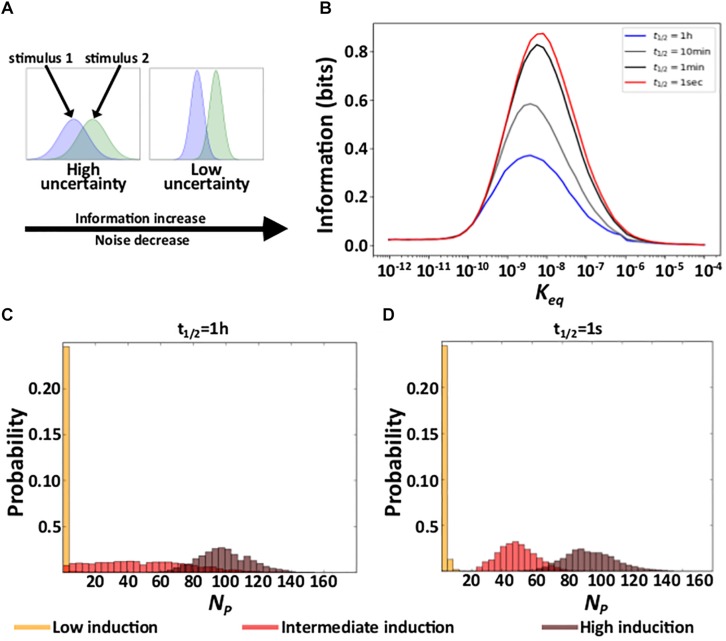
Residence time and information. **(A)** Schematic explanation of the relationship between noise and acquired information. The panel shows hypothetical response protein distributions produced by two different stimuli at a high (right) and a low (left) level of gene expression noise. **(B)** Acquired information at different residence times as a function of the affinity (*K*_*eq*_) between TF molecules and *DNA*. **(C,D)** Distributions of the number of expressed proteins *N*_*P*_ at three different TF concentrations (see color legend) with a long **(C)**, and a short **(D)** residence time. In **(C)** and **(D)**, *K*_*eq*_ = 10^–9^M.

Due to noise, the regulation of gene expression transforms a concentration of a TF into a distribution of expressed mRNA and protein molecules. Different TF concentrations may produce overlapping distributions of expressed molecules, in which case information about TF concentration gets lost. Reducing gene expression noise can reduce this overlap and thus also the amount of lost information (Figure 3A; Rhee et al., 2012). Because shorter residence times reduce gene expression noise (Figure 2), we hypothesized that they also increase the amount of information protein which expression levels contain about the concentration of the regulating TF. To find out, we quantified the mutual information between protein expression and TF concentration. Mutual information is an information theoretical quantity that encapsulates the reduction in uncertainty about one random variable provided by knowledge about another random variable (Cover and Thomas, 2006) (see “Materials and Methods”). To quantify mutual information, we performed 2500 stochastic simulations of gene expression dynamics for each of *n* evenly distributed TF concentrations within the interval [10^–7^M/*n*, 10^–7^M], exploring affinity values within the interval [10^–12^M, 10^–4^M]. This range includes affinity values below the minimal TF concentration, where induction is low regardless of TF concentration, and above the maximal concentration, where induction is high regardless of TF concentration.

In earlier work, we have shown that the amount of information that gene expression levels contain about the concentration of the regulating TF depends on the affinity between a TF and its binding site. At very low affinities, gene regulation is insensitive to TF concentration, such that gene expression conveys little information. At very high affinities, the level of gene induction is high for all TF concentrations, such that gene expression also conveys little information – it is similar to that of a constitutively expressed gene for all TF concentrations (Azpeitia and Wagner, 2019). Our current work shows that this pattern holds regardless of residence time (Figure 3B and Supplementary Figure 4A).

In contrast, residence time does affect acquired information at intermediate affinities. Specifically, even though the mean number of expressed molecules does not depend on a TF’s residence time on DNA (Figure 2E and Supplementary Figure 2E), their variability decreases with shorter residence times (Figure 2A). As a result, as residence time decreases, the overlap between protein distributions decrease (Figures 3C,D and Supplementary Figures 4B,C), which increases the amount of acquired information (Figure 3B and Supplementary Figure 4A). In sum, under conditions where a gene regulation system can acquire information, shortening residence time increases the amount of acquired information.

## Discussion

Previous theoretical and experimental work showed that gene expression noise can be modulated by the dissociation rate *k*_*d*_ of a DNA-bound TF (Peccoud and Ycart, 1995; Raser, 2004; Iyer-Biswas et al., 2009; Skupsky et al., 2010; Grönlund et al., 2013; Kumar et al., 2015; Fujita et al., 2016; Donovan et al., 2019), but this work did not distinguish between the effects of residence time and affinity. This distinction, however, is important because both properties depend on the dissociation rate but have different effects on gene expression dynamics. Here, using a stochastic model of gene activation by a TF, we study these properties separately, and show that short residence time can reduce expression noise, even though such noise generally increases when affinity decreases (Figure 2).

Our results also show that the effect of residence time on gene expression is not independent from affinity. The reason is that the level of induction depends on the affinity. When a gene is highly induced, residence time does not affect gene expression. However, as the level of induction decreases, short residence times can help produce less noisy expression. Short residence times effectively fragment gene activity into short periods of active and inactive expression, which prevents the excessive accumulation and depletion of proteins, and thus also excessive stochastic variation in gene expression. Similarly, the effect of affinity on noise depends on residence time. In particular, the effect of affinity is reduced when residence time decreases. However, noise can even decrease with decreasing affinity when residence times are very short and in the range of seconds (Figure 2A).

In previous work, we showed that expressed proteins harbor information about the concentration of a TF regulating their expression, if the TF’s affinity to regulatory DNA is of the same order of magnitude as its concentration (Azpeitia and Wagner, 2019). Here, we show that this behavior is independent of residence time. However, because shorter residence times render gene expression less noisy, the overlap between protein expression distributions resulting from different TF concentrations decreases. Consequently, shorter residence times increase the ability of a gene regulation system to distinguish between different TF concentrations.

Recent technological advances will permit experimental verification of our observations. First, in the last decade methods to quantify the ability of a regulated gene to acquire information about the concentration of a TF have become standardized (Cheong et al., 2011; Rhee et al., 2012). Also, the effect of residence time on noise can be quantified with techniques that simultaneously quantify TF-DNA binding events and the production of mRNA molecules, which are being developed (Donovan et al., 2019). It is especially difficult to obtain multiple alleles of a TF with different residence times without also altering the TF’s affinity to DNA. However, because our model shows that affinity affects gene expression by changing the induction level at a specific TF concentration, our observations hold regardless of whether one varies TF concentration or affinity (Supplementary Data 1). Hence, to test the effect of residence time on noise and information acquisition, one can compensate for any change in affinity by adjusting a gene’s induction level with TF concentration.

Our two state model of gene regulation is simple and does not explicitly represent all potentially relevant processes, such as the binding of RNA polymerases to DNA, or the assembly of the transcription initiation complex (Cramer, 2019). However, previous work has shown that the two state model produces similar gene expression dynamics as more complex models (Kumar et al., 2015), suggesting that our main results may hold for such models. Nevertheless, each additional transcriptional regulatory step requires time, which may constrain the amount of time that a TF must be minimally bound to DNA before it can affect gene expression (Corzo, 2006). For example, previous work has estimated that the binding of RNA polymerase takes on average 0.1 min, while the assembly of protein complexes takes between tens of second to minutes (Rybakova et al., 2015; Choubey et al., 2018). The results of our model hold as long as all these other processes have enough time to occur.

Future work will also need to consider other kinetic parameters affecting gene expression, such as the mRNA synthesis rate, because noise and information can be affected by these parameters (Peccoud and Ycart, 1995; Kepler and Elston, 2001; Raser, 2004; Tkacik et al., 2008; Raj and van Oudenaarden, 2009; Rieckh and Tkačik, 2014; ten Wolde et al., 2016). Moreover, many genes have a leaky or basal level of gene expression (Huang et al., 2015). Although we have not explicitly modeled leaky and basal expression, we believe that including leaky and basal gene expression in our model will not modify our results, because the effect of TF regulation on gene expression is independent from that of basal and leaky expression. However, previous work has shown that leaky expression can reduce noise (Huang et al., 2015). Further research is thus needed to evaluate the effect of leaky and basal gene expression.

We only studied TFs that activate gene expression. However, TFs can also inhibit gene expression. Independently of a TF’s effect on gene expression (i.e., activation or inhibition), long residence times will result in long periods of time when a TF is either bound to DNA or unbound. For a TF inhibiting gene expression, during the long periods where the TF is bound, gene expression will decay, while during the long unbound periods gene expression will increase. Hence, we believe that long residence times will still result in large gene expression fluctuations when a TF inhibits gene expression. For this reason, we believe that reducing residence time will decrease gene expression noise for both TF activation and inhibition. This hypothesis, however, still needs to be studied.

Our results are in agreement with previous work and complement this work. For example, experimental evidence suggests that the affinity of essential regulators of gene expression, such as NF-κB and TBP, modulates gene expression noise (Ravarani et al., 2016; Heltberg et al., 2019). Moreover, a model based on the binding dynamics of Sox2 and Oct4, two important regulators of the pluripotency of stems cells, showed that long residence times reduce the sensitivity of gene expression to TF concentration, because TFs with long residence times are bound to DNA most of the time regardless of their concentration (Chen et al., 2014; Liu and Tjian, 2018). Another study showed that negative regulatory feedback loops in general suppress noise more effectively when residence times are short (Grönlund et al., 2013).

Our work also helps solve an apparent contradiction between experimental and theoretical work about the importance of residence time. In particular, it has been predicted that longer residence times facilitate gene expression, because they increase the probability of a successful activation of gene expression by TFs, by providing longer time for other components, such as polymerases, to successfully bind DNA (Stavreva et al., 2004; Gorski et al., 2008; Lickwar et al., 2012; Mueller et al., 2013; Clauß et al., 2017). Experimentally measured residence times, which are generally short and lie within seconds to minutes (Hager, 2009; Geertz et al., 2012; Mazza et al., 2012; Gebhardt et al., 2013; Mueller et al., 2013; Chen et al., 2014; Morisaki et al., 2014; Sugo et al., 2015; Deluz et al., 2016; Clauß et al., 2017; Liu and Tjian, 2018; Rullan et al., 2018), are inconsistent with this prediction. Our work shows that residence time does not affect average gene expression levels. Instead, residence time reduces expression noise and can help signaling systems acquire information without modifying the probability of a successful activation of gene expression, which can help explain why short residence times may be prevalent in nature.

## Materials and Methods

### Two-State Model of Gene Expression

To study how a TF’s residence time on DNA affects gene expression, we built a gene expression model in which a *TF* binds to a DNA binding site (*DNA*_*bs*_) to regulate the expression of a nearby gene. *TF* molecules associate with the DNA binding site at a rate *k*_*a*_ (M^–1^s^–1^), and dissociate from it at a rate *k*_*d*_ (s^–1^). Only when the TF is bound to DNA does transcription occur [at a rate *k*_1_ (s^–1^)]. Transcribed mRNA molecules are degraded at a rate *d*_1_ (s^–1^). Proteins are translated from mRNA molecules at a rate *k*_2_ (s^–1^), and degraded at a rate *d*_2_ (s^–1^).

### Stochastic Simulations

To simulate the behavior of our gene expression model, we use Gillespie’s discrete stochastic simulation algorithm (Gillespie, 1977), using the numpy python package for scientific computing^1^. Gillespie’s algorithm captures the stochastic nature of chemical systems. It assumes a well-stirred and thermally equilibrated system with constant volume and temperature. The algorithm requires the probability *p*_*j*_ that a chemical reaction *R*_*j*_ occurs in a given time interval [t, t + τ]. This probability *p*_*j*_ is proportional to both the reaction rate and the number of reacting molecules. For the reversible bindings of TF molecules to DNA, the association probability *p*_*a*_ and the dissociation probability *p*_*d*_ are given by

$$p_{a}=\frac{k_{a}}{VN_{A}}N_{T}N_{D}$$

$$p_{d}=k_{d}N_{TD}$$

where *V* is the reaction volume, *N*_*A*_ is Avogadro’s number, and *N*_*T*_, *N*_*D*_, and *N*_*TD*_ are the numbers of TF molecules, DNA binding sites, and TF-*DNA*_*bs*_ complexes. Notice that the dissociation of TF-*DNA*_*bs*_ complexes is a first-order reaction, which is independent of the volume in which the reaction takes place. In contrast, the association of TF molecules with DNA binding sites is a second-order reaction, which is inversely proportional to the volume.

The probabilities *p*_*mRs*_, *p*_*mRd*_, *p*_*Ps*_, and *p*_*Pd*_ of mRNA transcription, mRNA degradation, protein synthesis, and protein degradation are given by

$$p_{mR_{s}}=k_{1}N_{TD}$$

$$p_{mR_{d}}=d_{1}N_{mR}$$

$$p_{P_{s}}=k_{2}N_{mR}$$

$$p_{P_{d}}=d_{2}N_{P},$$

respectively. In these expressions, the quantities *N*_*TD*_, *N*_*mR*_, and *N*_*P*_ are the numbers of TF-DNA complexes, mRNA molecules and of protein molecules, respectively. Because we model a haploid organism with only a single non-leaky DNA binding site, the probability of mRNA synthesis can be reduced to

$$p_{mR_{s}}=k_{1}$$

when the DNA is bound by the TF (*N_*TD*_* = 1), and to

$$p_{mR_{s}}=0$$

when it is unbound (*N_*TD*_* = 0).

### Initial Conditions for Simulations

We assume that a TF concentration of 10^–7^M corresponds to a few thousand TF molecules per cell, a realistic number in animal and yeast cells (Biggin, 2011; Ho et al., 2018). Because we model only one binding site, the concentration of free TF is not substantially affected by the binding of a single TF molecule to *DNA*. We therefore do not distinguish between the free and the total TF concentration. After this simplification, to determine the initial conditions of our model, we calculate the probability ${\hat{N}}_{TD}$ that the binding site is bound by a TF molecule as

$${\hat{N}}_{TD}=\frac{N_{T}}{K_{eq}+N_{T}}$$

where *N*_*T*_ is the total number of TF molecules. We selected the initial state of the DNA (*N*_*TD_i_*_) at random with binomial probability ${\hat{N}}_{TD}$ (i.e., *N*_*TD*_*i*__ = 1) if the DNA is bound by a TF molecule, and zero otherwise. It follows that

$$N_{D_{i}}=1-N_{TD_{i}}$$

$$N_{T_{i}}=N_{T}-N_{TD_{i}}$$

where *N*_*D_i_*_ is the initial state of the number of non-bound DNA binding site and *N*_*T_i_*_ is the number of free TF molecules. As the initial state of the number mRNA and protein molecules we used

$$N_{mR_{i}}={\hat{N}}_{TD}\frac{k_{1}}{d_{1}}$$

$$N_{P_{i}}={\hat{N}}_{TD}\frac{k_{1}}{d_{1}}\frac{k_{2}}{d_{2}},$$

which are the expected average number of mRNA and protein molecules for a constitutively expressed gene (Raj and van Oudenaarden, 2009), multiplied by the probability that the DNA is bound by a TF molecule.

### Information Quantification

The number of molecules of any chemical species in a cell or in a unit volume fluctuates, because molecules are produced and decay stochastically. We use Shannon’s entropy to quantify the unpredictability caused by such stochastic fluctuations in the number of TF molecules as

$$H\left(\text{Pr}\left(TF\right)\right)=-\sum_{N_{T}=\frac{N_{Tmax}}{n}}^{N_{Tmax}}p\left(N_{T}\right){\text{log}}_{2}p\left(N_{T}\right)$$

where Pr(*TF*) is the distribution of the number of transcription factor molecules (*N*_*T*_), and *p(N_*T*_)* is the probability that the system contains *N* molecules of the TF.

To estimate information we performed simulations from *n* different numbers of TF molecules that were evenly distributed within the interval [*N*_*Tmax*_/*n*,*N*_*Tmax*_] (*n* < *N*_*Tmax*_). For this reason

$$H\left(\text{Pr}\left(TF\right)\right)={\text{log}}_{2}n.$$

TF-DNA binding triggers the transcription of mRNA molecules that are then translated into protein molecules. We use the number of mRNA *N*_*mRNA*_ and protein molecules *N*_*P*_ as the system’s response or output, which we denote as *O*.

A gene expression system acquires information when the number of expressed proteins or mRNA reflects the number of TFs. This information can be quantified via the mutual information

I(TF;O)=H(Pr(TF))-H(Pr(TF|O)),

a widely used quantity in information theory (Cover and Thomas, 2006). It is equal to the difference between the entropy *H*(Pr(*TF*)) and the conditional entropy *H*(Pr(*TF*| *O*)), which represents the entropy of the number of TF molecules for a given number of mRNA or protein molecules. In other words, the mutual information *I* quantifies the amount of information that the number of expressed mRNA or protein molecules harbors about the number of TF molecules.

### Noise Quantification

The model produces a probability distribution of the number of mRNA and protein molecules for any given number of TF molecules *N*_*T*_. This response is thus a conditional probability distribution, which we write as

$$\text{Pr}\left(N_{O_{min}}\leq N_{O}\leq N_{O_{max}}|TF=N_{T}\right)$$

$$\text{Pr}\left(N_{Omin}<N_{O}<N_{Omax}|TF=N_{T}\right),$$

where *N*_*Omin*_ and *N*_*Omax*_ are the minimal and maximal number of mRNA or protein molecules, respectively. We performed 1000 simulations to estimate noise using three different measures. The size of the fluctuation, Fano factor and the coefficient of variation. The size of the fluctuations was quantified as the average difference (*N_O_max__*−*N_O_min__*). Fano factor as the variance of the response distribution divided by its mean (σ^2^(*N*_*P*_)/${\bar{N}}_{P}$). Coefficient of variation as the standard deviation of the response distribution divided by its mean (σ(*N*_*P*_)/${\bar{N}}_{P}$).

### Parameter Values

Our simulations considered biologically sensible parameter ranges. Specifically, for TF-*DNA*_*bs*_ binding, empirical data suggests that usually *K_*eq*_* < 10^–8^ and can reach picomolar (10^–12^M) or even smaller values (Corzo, 2006; Biggin, 2011; Li et al., 2011; Fisher et al., 2012; Belikov et al., 2015; Jung et al., 2016). Because experimental research has shown that residence time varies from seconds to tens of minutes (Geertz et al., 2012; Mueller et al., 2013; Morisaki et al., 2014), we used dissociation rates producing residence time within this interval [1 s, 1 h].

For mRNA, experimentally measured half-lives usually lie in the range of seconds to hours (Sharova et al., 2009; Schwanhäusser et al., 2011; Laalami et al., 2014; Lugowski et al., 2018). Protein half-lives are usually longer than mRNA half-lives (Moran et al., 2013) and lie between hours and days (Schwanhäusser et al., 2011; Toyama and Hetzer, 2013). Taking all this information into consideration we chose mRNA half-lives of ∼3.3 min, and protein half-lives of ∼1.5 h. We assumed that the ratio *k*_2_/*k*_1_ of the protein synthesis rate to the mRNA synthesis rate exceeds 1.0 (Hausser et al., 2019).

## Data Availability Statement

All datasets generated and analyzed for this study are included in the article/Supplementary Material.

## Author Contributions

AW and EA designed the project and wrote the manuscript. EA performed the simulations and analysis of the data.

## Conflict of Interest

The authors declare that the research was conducted in the absence of any commercial or financial relationships that could be construed as a potential conflict of interest.

## Footnotes

1 http://www.numpy.org/
