# Supplementary material for: Short Residence Times of DNA-Bound Transcription Factors Can Reduce Gene Expression Noise and Increase the Transmission of Information in a Gene Regulation System
Source: Front Mol Biosci. 2020 Apr 28;7:67. doi: 10.3389/fmolb.2020.00067 (PMC7198700; doi:10.3389/fmolb.2020.00067)
Supplement: Supplementary file 1 [file Data_Sheet_1.pdf]

# Supplementary Material

## 1 Supplementary Data

### Sup. Data 1. Affinity and induction level

As explained in the main text, the affinity of a TF to its DNA binding site and the TF concentration determine the induction level of a gene (Figure 1D). Hence, we hypothesized that the effect of affinity on gene expression could be reproduced by a change in the TF concentration. To find out, we repeated all our analyses, but instead of varying the TF concentration, we set it to a constant value of $10^{-9}$M. We then varied the affinity within the interval [$10^{-7}$M,$10^{-11}$M]. Analogous to our analysis in the main text, this interval includes Affinity values two orders of magnitude above and below the TF concentration. Hence, at the highest affinity, the level of induction is high and the regulated gene is almost always active. Conversely, at the lowest affinity, the level of induction is low and the regulated gene is almost always inactive. We were able to reproduce all observations we had made by varying TF concentration through the variation of the affinity. Most importantly, as the level of induction decreases, shorter residence times still reduce noise (compare Figure 2, Sup. Figure 1 and 2 with Sup Figure 5-7), producing a more homogenous and regular dynamic of gene expression (compare Sup. Figure 3 with Sup. Figure 8). Hence, our observations show that affinity affects gene expression dynamics mainly through its effect on gene induction.

### Sup. Data 2. Residence time and strength of noise

To study the effect of residence time and affinity on the level of gene expression noise, we also quantified the Fano factor, and the coefficient of variation of gene expression. The Fano factor is equal to the variance in the number of expressed molecules divided by its mean ($\sigma^2(N_P)/\overline{N}_P$), while the coefficient of variation is equal to the standard deviation divided by the mean ($\sigma(N_P)/\overline{N}_P$). Both measures provide information about the dispersion of the distribution of expressed molecules relative to its mean.

The Fano factor and the coefficient of variation display the same behavior as the standard deviation in the number of expressed molecules (i.e., the size of the fluctuations). When a gene is highly induced at the highest affinity value, residence time does not affect any of these quantities (Sup. Figure 1). As explained in the main text, the reason is that the regulated gene behaves like a constitutive gene in this case, where the fluctuations of protein concentrations around their mean do not depend on a TF's residence time (Figure 2A and D).

At the opposite extreme, as induction approaches zero, longer residence times increase the Fano factor and the coefficient of variation. The reason is that longer residence times produce larger fluctuations in the number of expressed molecules (Figure 2A). Not surprisingly then, the variance in protein number is higher for longer residence times, which increases the strength of noise (Sup. Figure 1), because the mean protein and mRNA expression is not affected by residence time (Figure 2E; Sup. Figure 2E). In sum, similar to our results in the main text, as the level of induction decreases, the amount of noise decreases with residence time.

**Sup. Data 3. Protein and mRNA production and degradation events**

Because shorter residence times (at submaximal induction) reduce variation in the frequency of protein production and degradation events, we hypothesized that production and degradation events should alternate more regularly at short residence times, such that the number of consecutive production events (i.e., the number of protein production events without any intervening degradation event should decrease). To validate this hypothesis, we quantified the number of consecutive production events at different levels of induction and residence times. When induction is high, residence time does not affect the number of consecutive production events (Sup. Figure 3C and E), because gene expression resembles that of a constitutive gene at all residence times (Figure 2A and B). However, as induction decreases, shorter residence times decrease both the mean and standard deviation number of consecutive production events (Sup. Figure 3C-F), showing that production and degradation events alternate more frequently.

One exception to this pattern occurs at the lowest level of induction. Here, the mean and standard deviation of the number of consecutive production events reaches a maximum at intermediate residence time and decreases as the residence time increases (Sup. Figure 3C-F, yellow). We believe that the reason is that most production events tend to occur in a few but long periods of active gene expression when the induction level is low and the residence time is long. During these long periods of active gene expression, protein expression behaves as for a highly induced gene, which has a smaller mean and standard deviation of the number of consecutive production events (Sup. Figure 3C-F). Notice, however, that this dynamic will still produce high levels of noise, because mRNA and protein molecules will fluctuate between very low and very high levels (Figure 2A; Sup. Figure 2A). In conclusion, the more frequent alternation of production and degradation events observed at shorter residence times produce a more regular and less noisy dynamic of expression.

## 2 Supplementary Tables

**Sup. Table 1**. Parameter values used for the simulations. $k_d$, $k_1$, $k_2$, $d_1$, $d_2$ units are $s^{-1}$. $k_a$ units are $M^{-1}s^{-1}$. The concentration of the TF is in M.

| | $k_a$ | $k_d$ | $k_1$ | $k_2$ | $d_1$ | $d_2$ | TF conc |
|---|---|---|---|---|---|---|---|
| Figure 2 Sup. Figure 1A, 1B, 3A, 3C and 3D | $[2.7*10^5, 10^8]$ | $[2.7*10^{-4},1]$ | 0.01 | 0.011 | 0.005 | 0.00022 | $[10^{-11},10^{-7}]$ |
| Figure 3 | $[2.7, 1.0^{12}]$ | $[2.7*10^{-4},1]$ | 0.01 | 0.011 | 0.005 | 0.00022 | $[10^{-11},10^{-7}]$ |
| Sup. Figure 1C, 1D, 2, 3B, 3E and 3F | $[2.7*10^5, 10^8]$ | $[2.7*10^{-4},1]$ | 0.01 | NA | 0.0001 | NA | $[10^{-11},10^{-7}]$ |
| Sup. Figure 4 | $[2.7, 1.0^{12}]$ | $[2.7*10^{-4},1]$ | 0.01 | NA | 0.0001 | NA | $[10^{-11},10^{-7}]$ |
| Sup. Figure 5A, 5B, 6, 8A, 8C and 8D | $[2.7*10^3, 10^{10}]$ | $[2.7*10^{-4},1]$ | 0.01 | 0.011 | 0.005 | 0.00022 | $10^{-9}$ |
| Sup. Figure 5C, 5D, 7, 8B, 8E and 8F | $[2.7*10^3, 10^{10}]$ | $[2.7*10^{-4},1]$ | 0.01 | NA | 0.0001 | NA | $10^{-9}$ |

## 2.1 Supplementary Figures

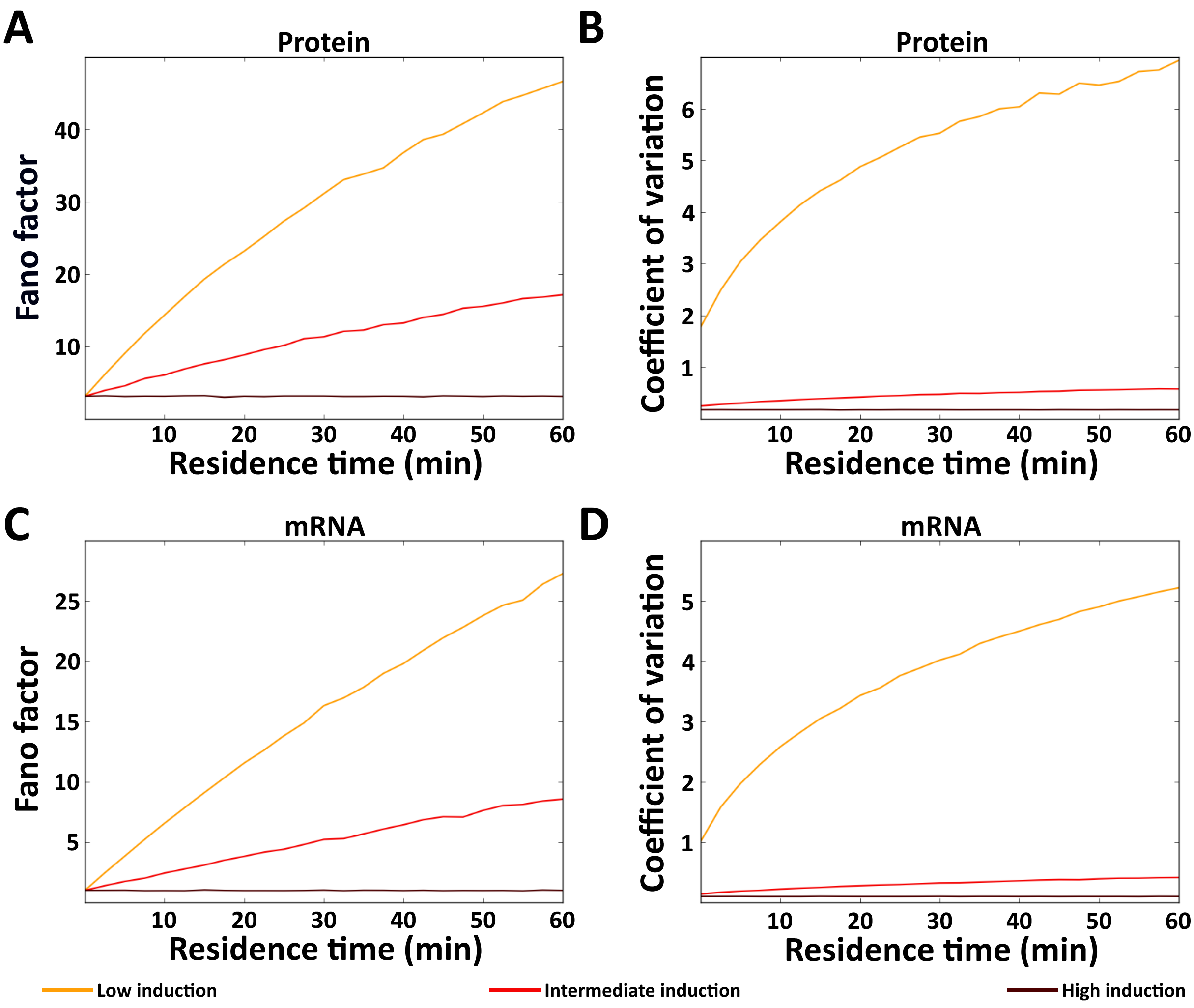


**Sup. Figure 1. Effect of residence time on Fano factor and coefficient of variation. (A and C)** Fano factor and **(B and C)** coefficient of variation in the number of expressed protein **(A and B)** and mRNA **(C and D)** molecules as a function of residence time (*x* axis) at a high (TF=$10^{-7}$M), intermediate (TF=$10^{-9}$M) and low (TF=$10^{-11}$M) levels of induction, as indicated by the color legend below the figure.

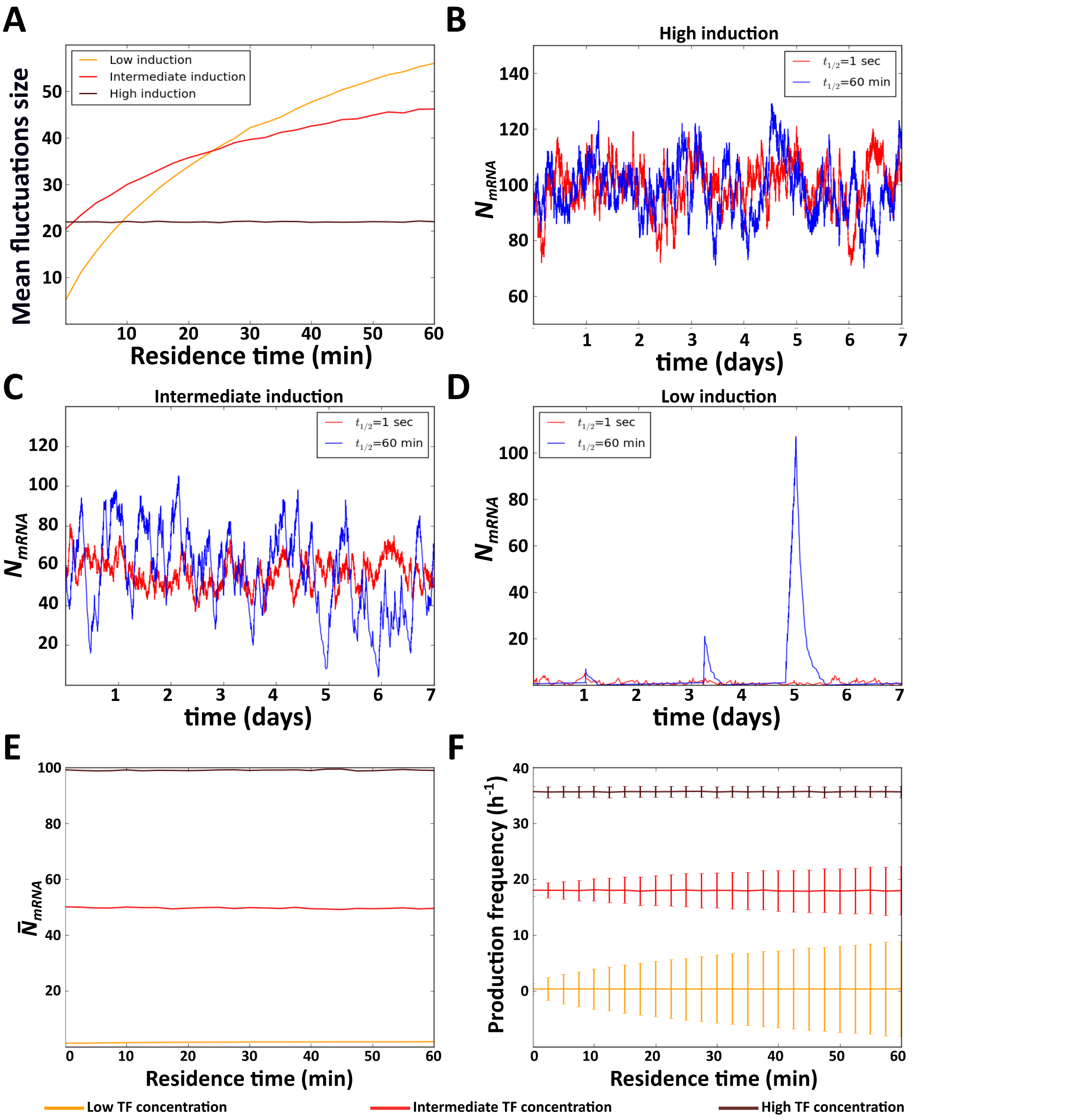


**Sup. Figure 2. Effect of residence time on the size of the mRNA fluctuations. (A)** Mean fluctuation size in the number of mRNA molecules ($y$ axis) at a high ($TF=10^{-7}M$), intermediate ($TF=10^{-9}M$) and low ($TF=10^{-11}M$) level of induction, as a function of residence time ($x$ axis). **(B-D)** Example time trajectories of the number of expressed mRNA molecules $N_{mRNA}$ obtained from the simulation of the model at three different levels of induction. **(B-D)** Red and blue lines show data for short (1s) and long (1h) residence times, respectively. Analyses of **(E)** mean number of mRNA ($\bar{N}_{mRNA}$) **(F)** mean and coefficient of variation of the frequency of mRNA production events at high ($TF=10^{-7}M$), intermediate ($TF=10^{-9}M$), and low ($TF=10^{-11}M$) TF concentrations, as indicated in the color legend below the figure.

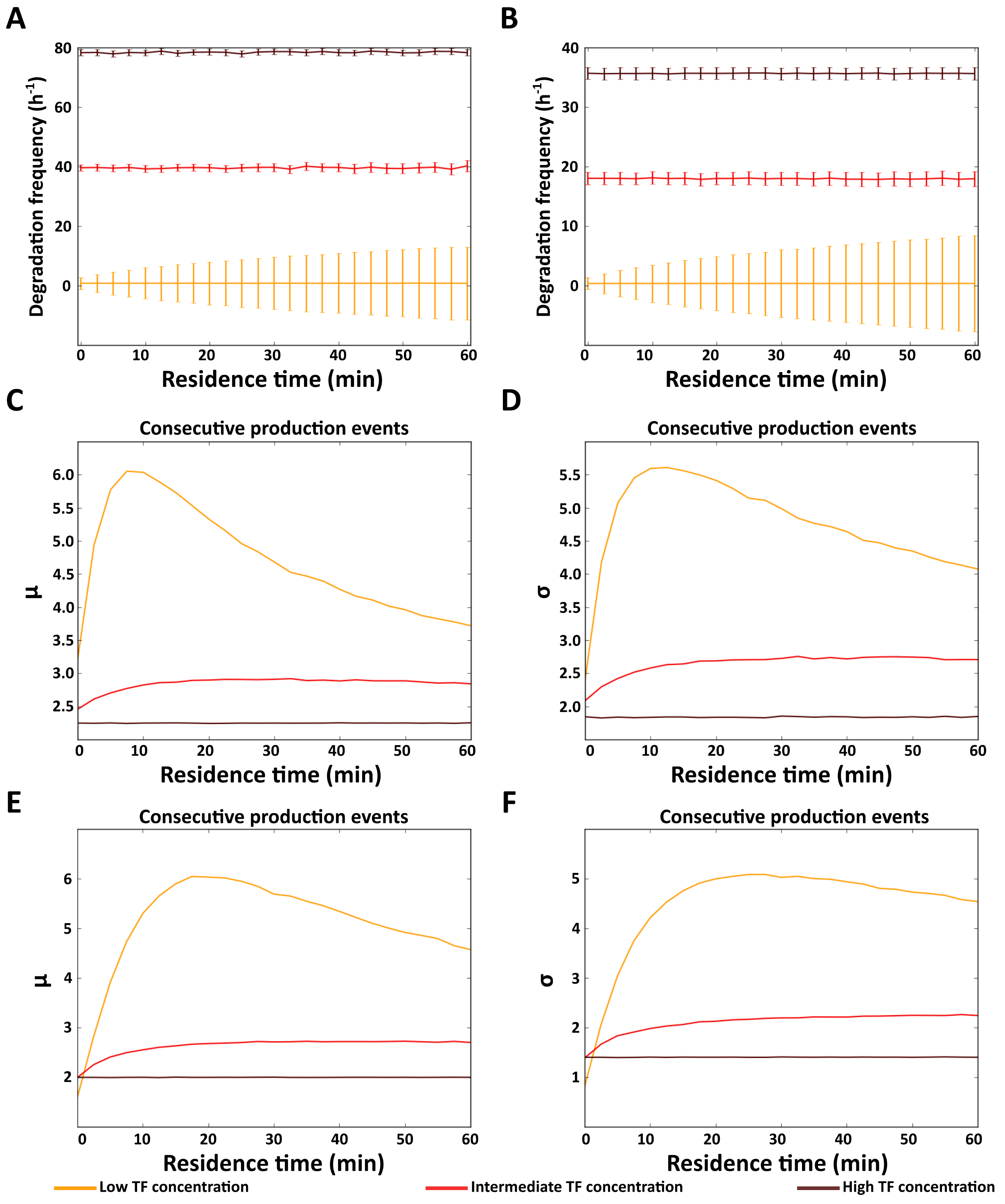


**Sup. Figure 3. Residence time and both production and degradation events.** Mean and standard deviation of **(A)** protein and **(B)** mRNA degradation events. **(C and E)** Mean and **(D and F)** standard deviation of the number of consecutive **(C and D)** protein and **(E and F)** mRNA production events as a function of residence time (*x* axes). All analyses were performed at high (TF=$10^{-7}$M), intermediate (TF=$10^{-9}$M) and low (TF=$10^{-11}$M) affinity values (see color legends).

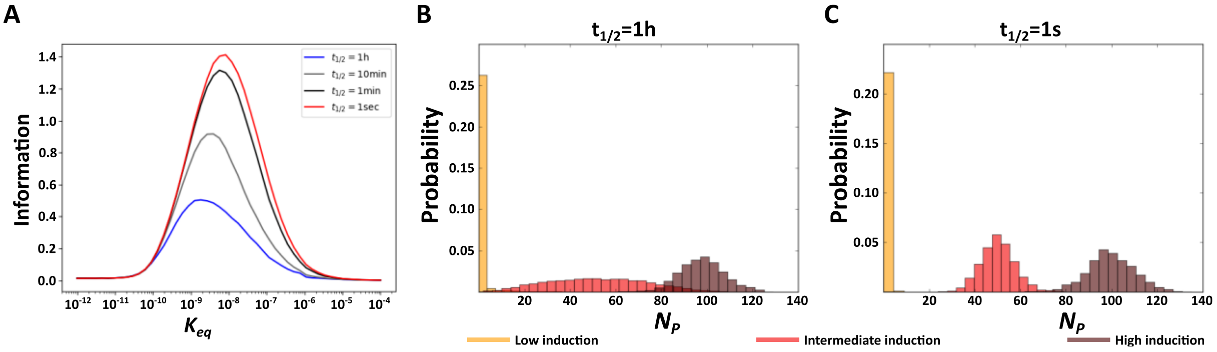


**Sup. Figure 4. Residence time and acquired information quantified through mRNA expression. (A)** Information acquired at different residence times as a function of the affinity ($K_{eq}$) between TF molecules and *DNA*. **(B and C)** Distributions of the number of proteins $N_P$ produced at three different TF concentrations with a long **(B)**, and a short **(C)** residence time. In **(C)** and **(D)**, $K_{eq}$ =$10^{-9}$M.

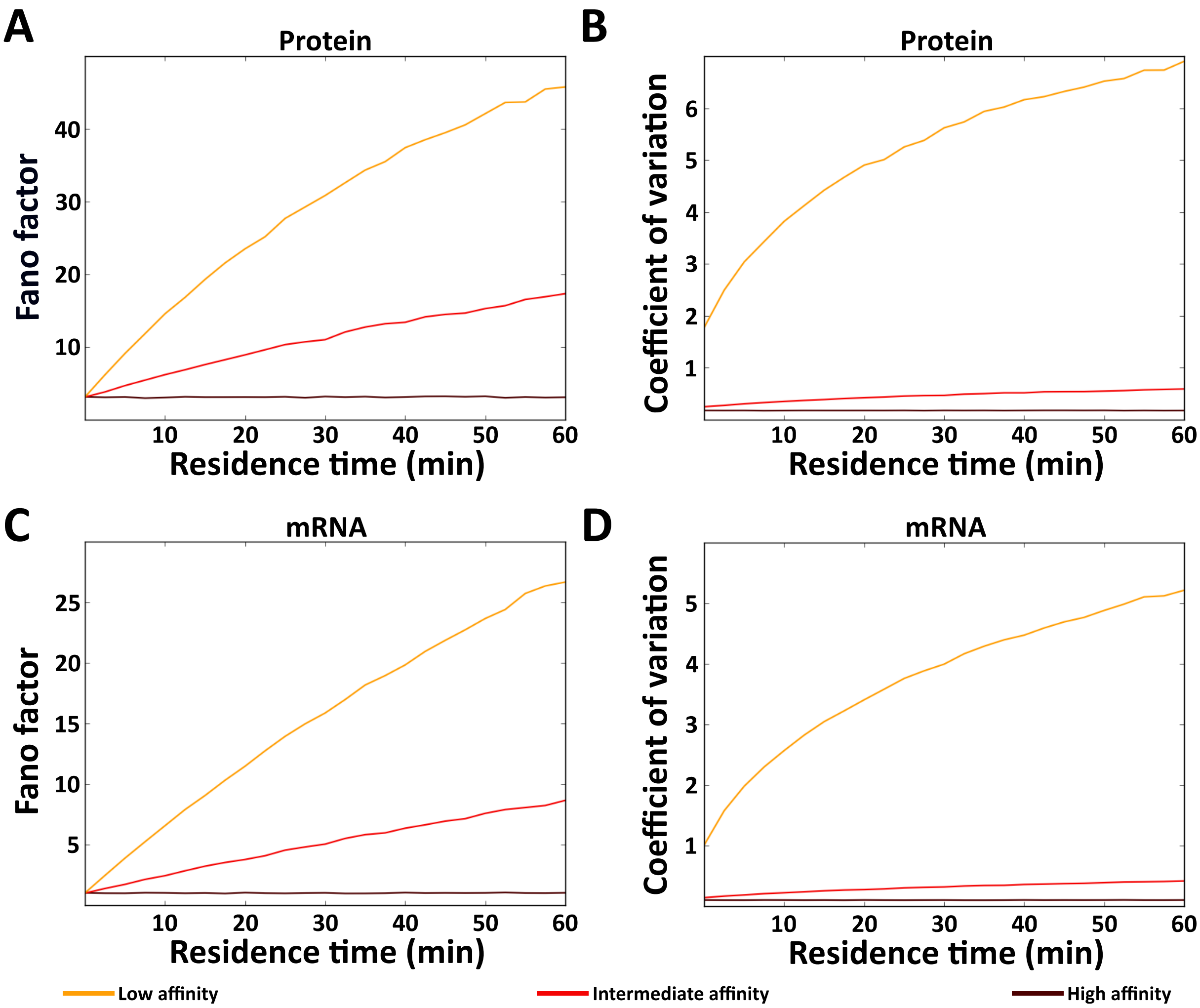


**Sup. Figure 5. Effect of residence time on Fano factor and coefficient of variation. (A and C)** Fano factor and **(B and D)** coefficient of variation in the number of protein **(A and B)** and mRNA **(C and D)** molecules as a function of residence time (*x* axes) at high ($K_{eq}$=$10^{-11}$M), intermediate ($K_{eq}$=$10^{-9}$M) and low ($K_{eq}$=$10^{-7}$M) affinity values, as indicated by the color legend below the figure.

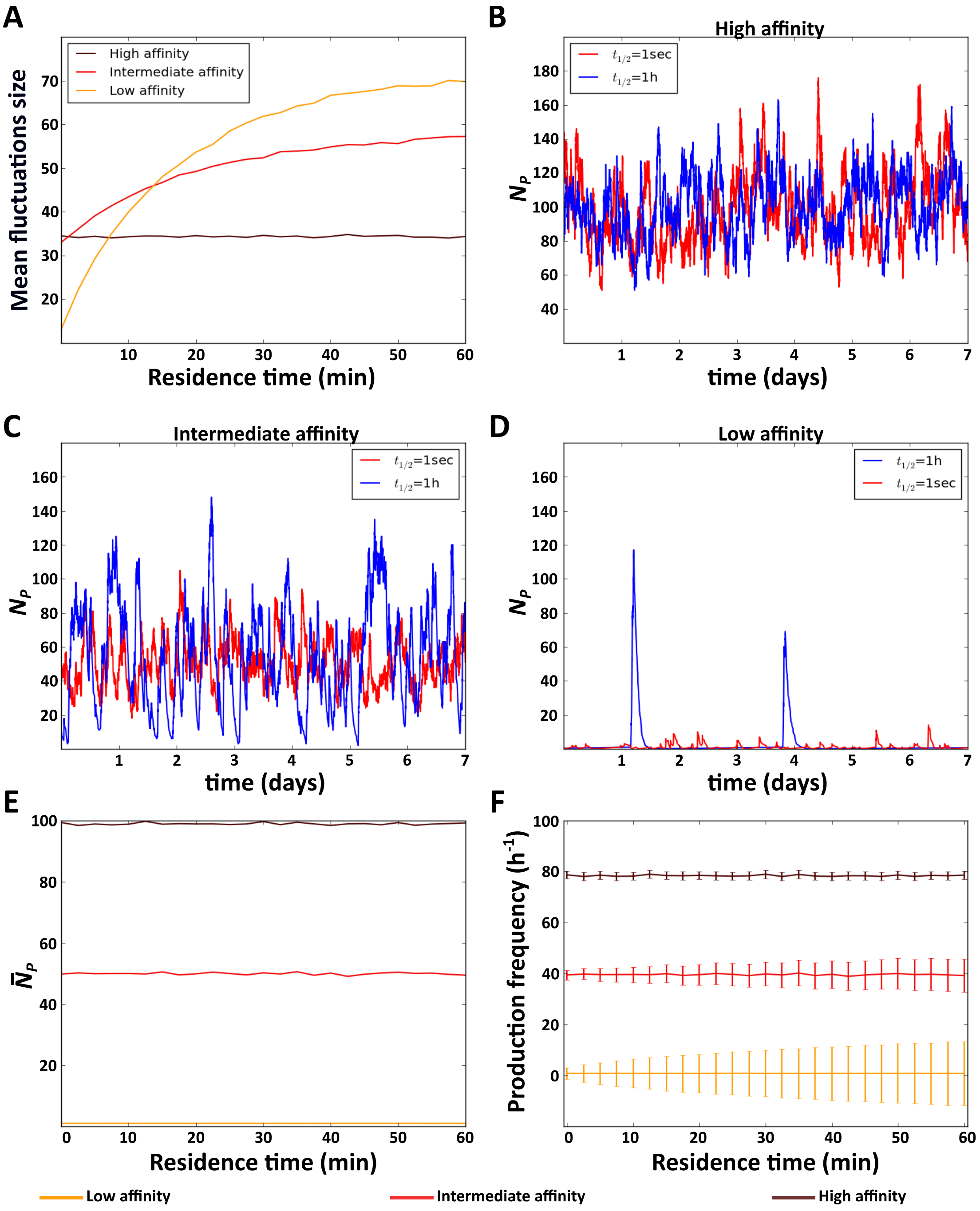


**Sup. Figure 6. Effect of residence time on the expression dynamic of protein molecules. (A)** Mean fluctuation size in the number of protein molecules (*y* axis) at high ($K_{eq}$=10$^{-11}$M), intermediate ($K_{eq}$=10$^{-9}$M) and low ($K_{eq}$=10$^{-7}$M) affinities, as a function of residence time (*x* axis). **(B-D)** Example time trajectories of the number of protein molecules $N_P$ obtained from the simulation of the model at the three different affinities. **(B-D)** Red and blue lines show data for short (1s) and long (1h) residence times, respectively. Analyses of **(E)** mean number of proteins ($\bar{N}_P$), and **(F)** mean and coefficient of variation of the frequency of protein production events.

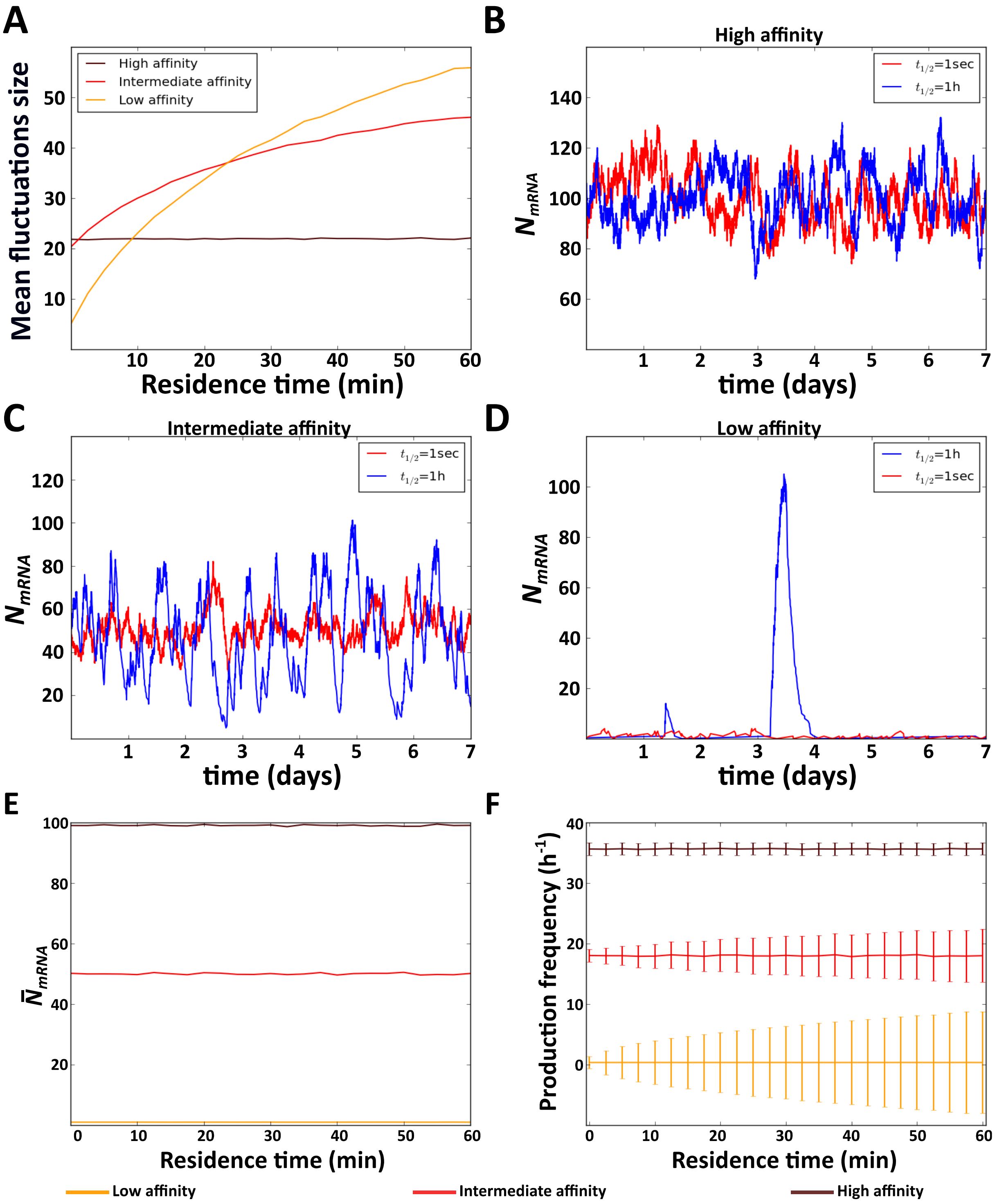


**Sup. Figure 7. Effect of residence time on time on the expression dynamic of mRNA molecules. (A)** Mean fluctuation size in the number of mRNA molecules ($y$ axis) at high ($K_{eq}$=$10^{-11}$M), intermediate ($K_{eq}$=$10^{-9}$M) and low ($K_{eq}$=$10^{-7}$M) affinity values, as a function of residence time ($x$ axis). **(B-D)** Examples time trajectories of the number of expressed mRNA molecules $N_{mRNA}$ at three different affinity values. **(B-D)** Red and blue lines show data for short (1s) and long (1h) residence times, respectively. Analyses of **(E)** mean number of expressed mRNA molecules ($\bar{N}_{mRNA}$), and **(F)** mean and coefficient of variation of the frequency of mRNA production events.

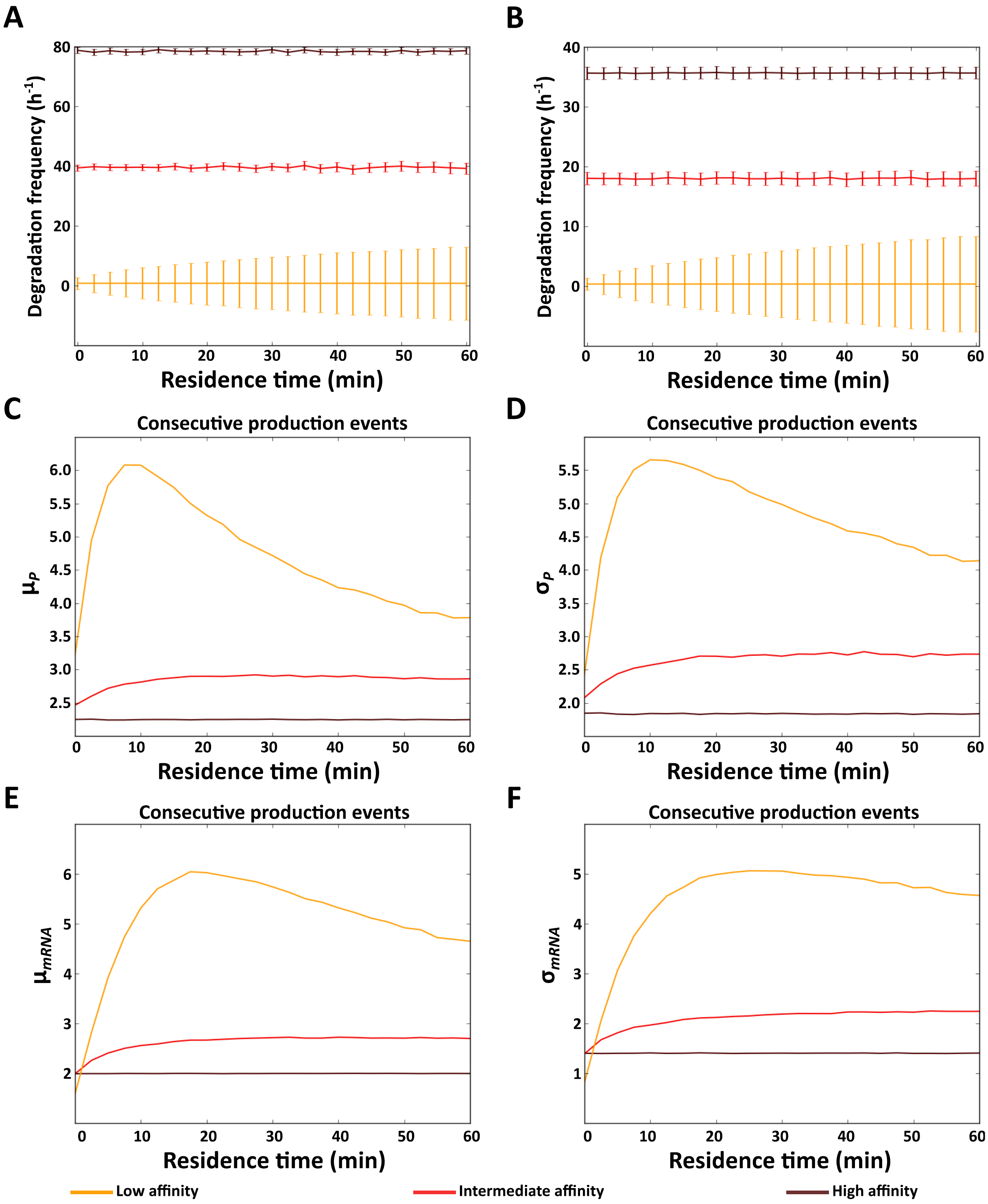


**Sup. Figure 8. Residence time and both production and degradation events.** Mean and standard deviation of **(A)** protein and **(B)** mRNA degradation events. **(C and E)** Mean and **(D and F)** standard deviation of the number of consecutive **(C and D)** protein and **(E and F)** mRNA production events as a function of residence time (*x* axes). All analyses were performed at high ($K_{eq}$=$10^{-11}$M), intermediate ($K_{eq}$=$10^{-9}$M) and low ($K_{eq}$=$10^{-7}$M) affinity values (see color legends).
